# Study on the Apoptosis Mechanism Induced by T-2 Toxin

**DOI:** 10.1371/journal.pone.0083105

**Published:** 2013-12-26

**Authors:** Zhenhong Zhuang, Daibin Yang, Yaling Huang, Shihua Wang

**Affiliations:** Key Laboratory of Pathogenic Fungi and Mycotoxins of Fujian Province, Key Laboratory of Biopesticide and Chemical Biology, Ministry of Education, and School of Life Sciences, Fujian Agriculture and Forestry University, Fuzhou, China; University of Pecs Medical School, Hungary; Jr

## Abstract

T-2 toxin is known to induce apoptosis in mammalian cells. The mechanism of apoptosis induced by T-2 toxin has been proposed to be linked with oxidative stress and mitochondrial pathway. In the current study, the toxic effect of T-2 on Hela, Bel-7402, and Chang liver cells was examined in dose-dependent and time-dependent manner by MTT assay. Caspase-3 was found to be up-regulated under T-2 toxin stress, which suggested that T-2 toxin induced cell apoptosis. Endogenous GSH and MDA levels in all three cell lines were found down- and up-regulated respectively, which indicated the link between toxic effect of T-2 toxin and intracellular oxidative stress. It was also found by MTT assay that NAC, which maintained the level of GSH in cells, could protect cells from death. Western-blot result showed that the level of both activated Caspase-8 and Caspase-9 increased when cells were treated by T-2 toxin. Caspase-9 was found to be activated earlier than Caspase-8. It was also found that p53 was up-regulated under T-2 toxin stress in the study. These results implied that the effect of T-2 toxin on cells was apoptosis rather than necrosis, and it was probably induced through mitochondrial pathway. To the best of our knowledge, the present study is the first to show that JunD is down-regulated in T-2 toxin induced apoptosis. By construction of an over-expression vector for the JunD gene, we observed that the survival ratio of JunD over-expressed cells obviously increased under T-2 toxin stress. These results suggested that the mechanism of T-2 induced cell death was closely connected with oxidative stress, and that JunD plays an important role in the defensive process against T-2 toxin stress.

## Introduction

T-2 toxin, a fungal secondary metabolite, is one of the type A Trichothecenes [1], [2]. Ingestion by humans or livestock of cereals contaminated by T-2 toxin can cause adverse reactions, such as vomit, diarrhea, and even death [3], [4]. Alimentary toxic aleukia (ATA), mainly due to ingestion of cereal containing large amounts of T-2 toxin has been reported to cause the death of a large number of people [5]. Injection of large dose of T-2 toxin to rat caused cardiomyopathy, which was similar to the symptom of ATA [6], [7]. In view of the great harm to the health of human and livestock, the toxicological effects of T-2 toxin was reported in the Joint Food and Agriculture Organization/World Health Organization (FAO/WHO) Expert Committee on Food Additives [4], [6]–[8].

It was reported that T-2 toxin could affect protein synthesis by its affinity with trans-peptidase, one of the important subunits in ribosome, and the biosynthesis of DNA and RNA were also inhibited by T-2 toxin [9], [10]. It was also found that T-2 toxin could interfere with the cytomembrane phosphorylation and cause lipid peroxidation in liver [11]. Islam *et* *al.* (1998) reported that the effect of T-2 toxin on mice thymocytes was apoptosis [12]. Shinozuka *et* *al.* (1997) also found that the T-2 toxin induced lymphocyte death was by apoptosis [13]. It was confirmed by in situ hybridization that the apoptotic process was accompanied by DNA damage [14]. Wang *et* *al*. (2012) further reported that JAK/STAT (Janus kinase/signal transducers and activators of transcription) might play an important role in the trichothecenes induced apoptosis [15]. T-2 toxin mainly acted on the metabolically active organs, such as spleen, thymus, marrow, stem cells, and so on [4], [16], [17]. The apoptotic process is initiated by a series of oxidative stress, and subsequently cells enter the mitochondrial death pathway [18], [19]. It was reported that T-2 toxin not only could down-regulate intracellular reduced GSH (Glutathione), but also could up-regulate intracellular total ROS (Reactive oxygen species) [5], which showed intrinsic link between T-2 toxin-induced apoptosis and oxidative stress. But the mechanism of how oxidative stress induces apoptosis is still unclear.

JunD, a member of the AP-1 family of transcription factors, regulates genes involved in antioxidant defense. Gerald *et* *al.* (2004) found that JunD could reduce angiogenesis in tumor by reducing ROS, and demonstrated that JunD involved in regulation of antioxidant defense [20]. Toullec *et* *al.* (2010) took advantage of JunD deletion cell strain to examine the role of ROS in tumor development, and uncovered the role of JunD in the suppression of the migratory properties of stromal fibroblasts, which in turn potentiate tumor dissemination [21]. However, there are still not any reports on the effect of JunD in the process of apoptosis induced by T-2 toxin.

In view of the harmful effects of T-2 toxin, this study focused on the mechanism of T-2 toxin-induced apoptosis, and on the role of oxidative stress, especially the function of JunD, in T-2 toxin induced apoptotic process.

## Materials and Methods

### Ethics Statement

All animal work was performed according to relevant national and international guidelines. All animal experiments were complied with the rules by the Animal Ethics Committee of the Fujian Agriculture and Forestry University.

### Materials

T-2 toxin was purchased from Sigma Corporation (USA),and caspase-3 colorimetric assay kit and MTT (3-(4, 5-Dimethylthiazol-2-yl)-2, 5-diphenyltetrazolium) assay kit were from Nanjing Keygen Biotech Co. Ltd (China). Lipofectamine 2000, anti-caspase-3 antibody, anti-caspase-8 antibody, anti-caspase-9 antibody, anti-JunD antibody, GSH assay kit, and MDA (Malondialdehyde) assay kit were from Beyotime Institute of Biotechnology (China). The other chemical reagents used were of analytical grade. *E. coli* BL21 (DE3) and DH5α, and interference vector pSliencer4.1 were preserved in our lab. Cell lines (Hela, Bel-7402, and Chang liver cells) were purchased from a typical cell culture collection Committee of the Chinese Academy of Sciences Library, and cultured in RPMI medium 1640 supplemented with 10% FBS (fetal bovine serum, Biotechnology Ltd. Co., Shanghai, China) [22].

### Inhibition Effect of T-2 Toxin on Cells

Cells (Hela, Bel-7402, and Chang liver) in logarithmic growth phase were transferred into 96-well plate (10^6^ cells per well, the cell density in the following experiments was the same), and were cultivated overnight. Then, 100 µL of T-2 toxin of various concentration (2000, 1000, 500, 250, 125, 62, and 30 ng/mL) was added respectively. DMSO (Dimethyl sulfoxide), the solvent for T-2 toxin, was added as control. After the cells were incubated with T-2 toxin for 24 h, MTT was added. Finally, the OD_570_ value was detected with microplate reader. The survival rate was calculated with the control as the reference. The lgIC50 of different cell lines was detected by the instruction of MTT assay kit: lgIC50 = Xm–I*(P−0.5), Xm: maximum dose, I: maximum dose/adjacent dose, P: the sum of all mortality. Inhibition rate = (1–OD value of test group)/OD value of control group.

### Detection of the GSH and MDA under T-2 Toxin Stress

Cells in logarithmic growth phase were transferred into 96-well plate, and were cultivated overnight. In every well, 1.6 mL T-2 toxin at a concentration of LC50 (50% lethal concentration) was added, and the cells were cultivated for 0, 4, 8, 16, 24 h respectively. After the incubation, cells were washed twice by PBS, the cells were mixed with protein removal reagent (S solution) at an amount of three times volume of cell pellet, and fully shocked by vortex. Following twice rapid freezing in liquid nitrogen and thawing in 37°C water bath, the samples were keep on ice for 5 min and centrifuged at 10000 g for 10 min under 4°C. At last, the volume of endogenic GSH was detected following the instruction of GSH extract and assay kit, and the volume of endogenic MDA was detected following the instruction of MDA extract and assay kit.

### Effect of NAC on Cells’ Viability and GSH Level under T-2 Toxin Stress

Cells in logarithmic growth phase were transferred into 96-well plate, and were cultivated overnight. In every well 100 µL 5 µmol/L NAC (N-acetyl cysteine) was added and incubated for 4 h. After the cells were washed twice by PBS, every well was treated with 100 µL T-2 toxin at a concentration of LC50, and cultivated for 6, 12, 18, 24 h respectively. Following steps referred to the methods mentioned in MTT assay kit and GSH detection and assay kit.

### Detection of Caspase-3, 8, 9, p53, and JunD by Western-blot Analysis

Cellular cultivation and T-2 toxin treatment was the same to that mentioned above. The cells in 96-well plate were cultivated for 8, 16, 24 h respectively. After the cells were collected and washed twice by PBS, cells were lysed for 3 min in 100 µL lysis buffer, then were centrifuged at 1000 g for 10 min, and the supernatant was kept on ice for further Western-blot analysis. Samples were used for SDS/PAGE on 12% gels by discontinuous buffer system at 15 mA. Proteins from the gels were transferred to Nitrocellulose (NC) membranes for 1 h at 60 V in transfer buffer (48 mM Tris, 39 mM glycine and 20% methanol) at 4°C. The membranes were incubated with corresponding antibody at a dilution of 1∶1000 in TNT buffer (1.211 g Tris, 8.77 g NaCl and 500 mL tween-20 in 1 L TNT, pH 7.0) containing 5% skim milk for 1 h at room temperature on a gentle shaker. The membranes were rinsed three times for 10 min with TNT buffer and incubated with goat anti-mouse HRP-conjuaged IgG at a dilution of 1∶4000 in TNT buffer containing 5% skim milk for 1 h at room temperature. The membranes were developed with substrate (ECL, Electrochemiluminescence) until optimum color developed [23].

### Effect of Over-expressed JunD on Cells’ Viability under T-2 Toxin Stress

The recombinant plasmid (JunD-PCDNA3.0, constructed previously in our lab) was extracted from *E. coli* DH5a, and transfected into cells by liposome (Lipofectamine 2000). After screening by G418, the JunD expression level was detected by Western-blot analysis with anti-JunD antibody as the first antibody [23]. The survival rate was calculated according to that mentioned in MTT assay.

### Statistical Analysis

The data from this experiment was analyzed by statistic software (SPSS 13.0), and all data was presented on the form of mean ± standard deviation (x ± s). The comparison between each group was based on single-factor analysis of variance. The comparison between parallel groups was analyzed with LSD test. The assessment of statistical significance of differences was carried out with one way ANOVA in Microsoft Excel. P<0.05 means that the differences are statistically significant.

## Results

### Dose and Time Dependent Cytotoxicity of T-2 Toxin to Cells

**Figure 1:**
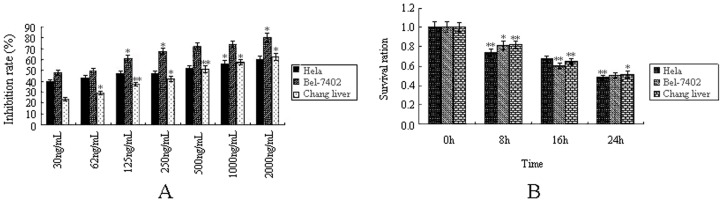
The inhibition effect of T-2 toxin on three cell strains. **A**. Dose-dependent inhibition ration of T-2 toxin at 24 h. **B**. Time-dependent effect of T-2 toxin to cells. Data was presented as mean ± SD. *p<0.05, **p<0.01, ***p<0.001.

MTT assay was used to detect the inhibition effect of T-2 toxin on cells. It could be concluded from Figure 1A that T-2 toxin was toxic to three lines of cells (Hela, Bel-7402, and Chang liver cell), and the virulence of T-2 toxin was different towards different cells. With the increase of the toxin dose, the cell mortality was increased, too. According to the method provided above, LC50 of T-2 toxin at 24 h to three cell lines (Hela, Bel-7402, and Chang liver cells) was found to be 357, 63, and 412 ng/mL respectively. The concentrations of T-2 toxin used in subsequent experiments were the LC50 of T-2 toxin presented above. Time effect was also observed when cells were stressed under LC50 of T-2 toxin. It could be found from the Figure 1B that as the time of T-2 stress extended, the survival rates of all three cell lines were decreasing obviously. It indicated that the effect of T-2 toxin on cells survival rate was not only dose-dependent, but also time-dependent.

### The Effect of T-2 Toxin on Endogenous GSH and MDA

**Figure 2:**
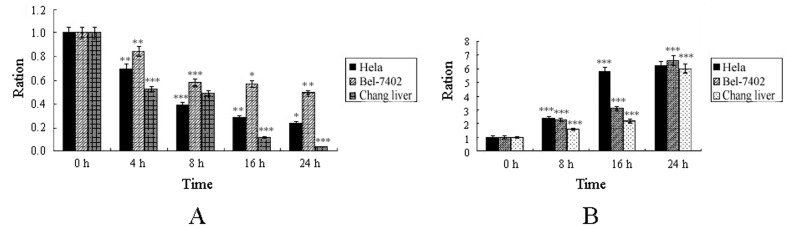
Intracellular redox level under T-2 toxin. A. Level of endogenous GSH under T-2 toxin stress. B. Levels of endogenous MDA under T-2 toxin stress. Data was presented as mean ± SD. *p<0.05, **p<0.01, ***p<0.001.

Endogenic GSH is an important antioxidant in cells, and its level impacts on cell anti-oxidative capacity directly. The decrease in GSH level would initiate intracellular oxidative stress. It could be found from Figure 2A that with the elongation of T-2 toxin treatment time, endogenic GSH in these cells was gradually reduced, and the endogenous GSH in test groups was less than half of that in the control group at 24 h. This indicated that the level of endogenous GSH would decrease when The cells were induced by T-2 toxin, and certain degree of oxidative stress took place in the cells. This was a hint for us to explore the level of MDA, a product of lipid peroxidation. The level of MDA inside cells usually increases significantly, when the cells are under oxidative stress. As shown in Figure 2B, all three cell lines produced different levels of MDA under T-2 toxin stress, and the level of MDA was gradually increased with the elongation of the treatment time. These results and previous reports showed that there was a link between the T-2 toxin stress and intracellular oxidative stress.

### The Protective Effect of Antioxidant NAC to the Cells against T-2 Toxin Stress

**Figure 3:**
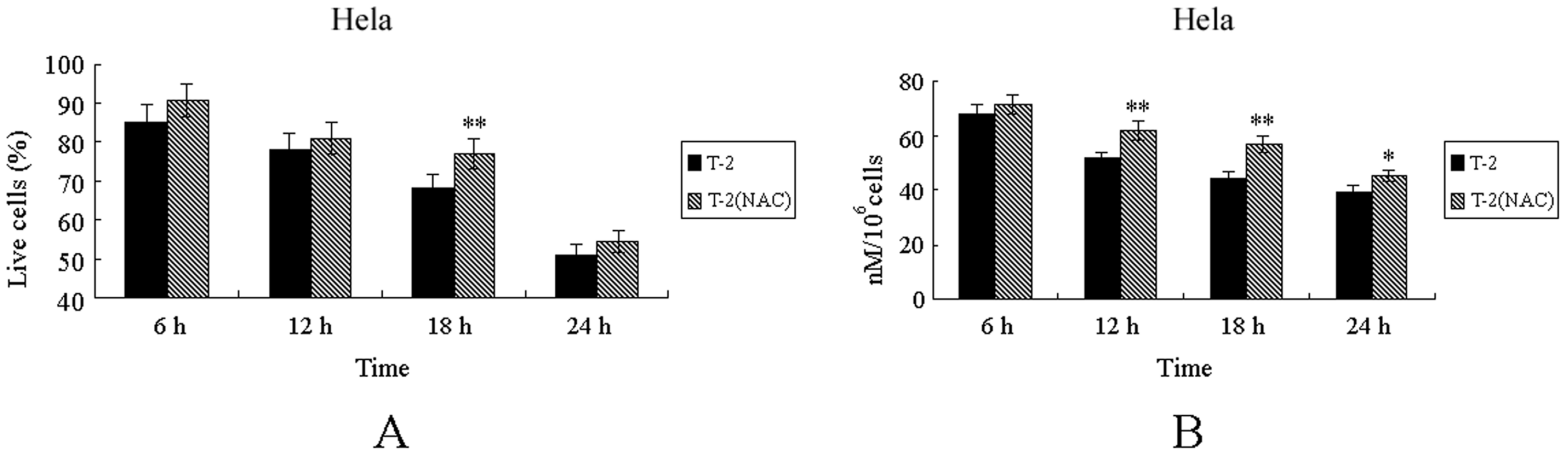
Antioxidation of NAC in cell death induced by T-2 toxin. **A**. Antioxidation of NAC in the cell death process of Hela cells. **B**. Endogenous GSH level of Hela under protection of NAC. Data was presented as mean ± SD. *p<0.05, **p<0.01, ***p<0.001.

In the study, antioxidant NAC was employed to test if it had protective effect on cells stressed by T-2 toxin. It could be found from Figure 3A that the survival rate of NAC pretreated Hela cells increased compared to the control group, which meant that the antioxidant NAC could relief the virulence of T-2 toxin, and alleviate T-2 toxin induced cell death. Endogenous GSH was detected, and the result from Figure 3B showed that there was more GSH in NAC pretreated Hela cells. But no obvious protective effect of NAC to the Chang liver and Bel-7402 cell lines under T-2 toxin stress was observed (data no shown). These results suggested that the pernicious effect of T-2 toxin on cells was at least partially caused by oxidative stress, and the protective effect of NAC to Hela cells against T-2 toxin stress was through protecting GSH.

### The Effect of T-2 Toxin Stress on Caspase-3

**Figure 4:**
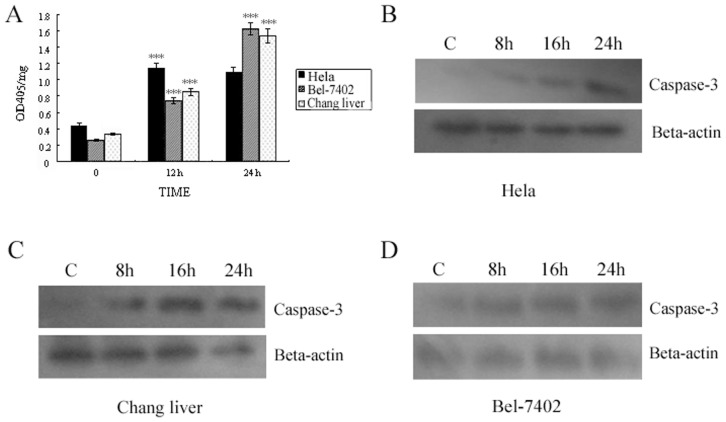
Activation of Caspase-3 induced by T-2 toxin. **A**. Detection of Caspase-3 hydrolase activity under T-2 toxin stress. **B**. Western blot result of Caspase-3 activated fragments in Hela cells when treated with T-2 at the concentration of LC50 for 8, 16, and 24 h respectively. **C**. Western blot result of Caspase-3 activated fragments in Changliver cells. **D**. Caspase-3 activity in Bel -7402 cell. Data was presented as mean ± SD. *p<0.05, **p<0.01, ***p<0.001.

Caspase 3, an important factor in apoptosis, is activated in both death ligand and mitochondrial pathways. In cell, Caspase 3 is an enzyme precursor that is activated only when cell initiates an apoptotic process. In the study, the hydrolase activity of Caspase 3 was detected to reflect the activity level of Caspase-3 in cells (Hela, Bel-7402, and Chang liver) under T-2 toxin stress. It could be observed from Figure 4A that the activity of Caspase-3 in three cell lines under T-2 toxin stress was increased 2–7 times compared to control group at 24 h. These results reflected that Caspase-3 played an important role in the process, and preliminary suggested that the process induced by T-2 toxin in cells was apoptosis.

The hydrolyzed or activated fragment of Caspase-3 was further analyzed by Western-blot analysis. The result showed that the level of activated hydrolysis fragment increased in all three cell lines (Figure 4 C–D). These results illuminated the effect of T-2 toxin to cells was apoptosis rather than necrosis.

### T-2 Toxin Induced Apoptosis by Mitochondrial Pathway

**Figure 5:**
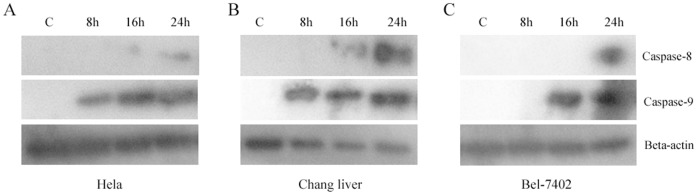
Western-blot analysis on activated Caspase-8 and Caspase-9 level. **A**. Activated Caspase-8 and Caspase-9 level in Hela cells when treated with T-2 toxin at the concentration of LC50 for 8, 16, and 24 h respectively. **B**. Activated Caspase-8 and Caspase-9 level in Changliver cells were treated with T-2 toxin. **C**. Bel -7402 cells were treated with T-2 toxin.

Members of the Caspase family are critical in the process of apoptosis. Caspase-8 mainly takes part in the death receptor pathway, and Caspase-9 is primarily working in mitochondrial pathway. The results of our Western-blot analysis (Figure 5) in present study showed that Caspase-8 was hydrolyzed and activated at about 16 h, and Caspase-9 was activated at about 8 h (Figure 5A and B). The same situation happened to the line of Bel-7402, and Caspase-8 was hydrolyzed and activated relatively later than that of Caspase-9. Caspase-8 was activated at about 24 h, but Caspase-9 was about 16 h (Figure 5C). These results indicated that the apoptosis induced by T-2 toxin was probably through mitochondrial pathway, which coincided with the previous reports [24], [25].

### The Effect of T-2 Toxin on the Level of p53

**Figure 6:**
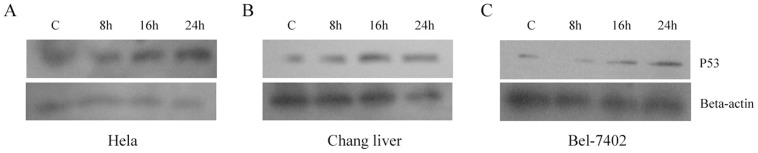
Levels of p53 under T-2 toxin stress. **A**. Levels of p53 in Hela cells when treated with T-2 at the concentration of LC50 for 8, 16, and 24 h respectively. **B**. Levels of p53 in Changliver cells. **C**. Bel-7402 cells were treated with T-2 Toxin.

The role of p53 in T-2 toxin induced apoptosis has been controversial. Some reports declared that the level of p53 did not change in the T-2 toxin mediated apoptosis [26], other reports found that p53 level was up-regulated in the process [25]. So it is necessary to further clarify the role of p53 played in the apoptotic process. We found that the protein level of p53 in these three cell lines (Hela, Chang liver, and Bel-7402) was up-regulated under T-2 toxin stress (Figure 6 A–C) suggesting that p53 took part in the apoptotic process induced by T-2 toxin.

### The Effect of Up-regulated JunD on Cells under T-2 Toxin Stress

**Figure 7:**
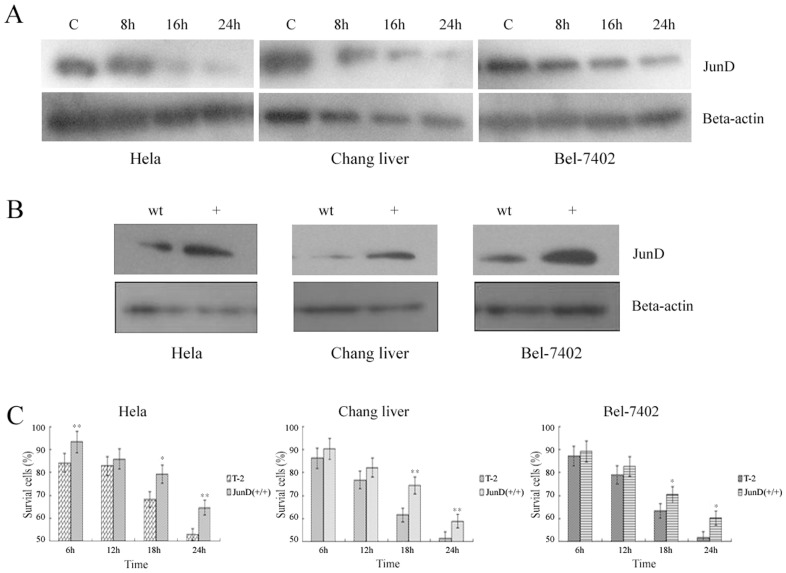
The effect of up-regulated JunD on cells. **A**. The expression levels of JunD induced by T-2 toxin. **B**. The expression of JunD after transfection of JunD over-expression vector in three cell strains. wt: wild type. +: cell strains transfected with over-expression vector. **C**. The effect of over-expressed JunD on cell survival rate. T-2: wild-type cells under T-2 toxin stress. JunD (+/+): JunD over-expressied cell lines under T-2 toxin stress. Data was presented as mean ± SD. *p<0.05, **p<0.01, ***p<0.001.

JunD plays a role in intracellular antioxidant system. In the present study, we explored the relationship between JunD and apoptosis induced by T-2 toxin. By Western-blot analysis, we found that the protein levels of intracellular JunD were obviously suppressed under T-2 toxin stress in all three cell lines we used **(** Figure 7A **)**. The down-regulation of JunD results in the suppression of intracellular antioxidant system, which makes the cells susceptible to oxidative stress.

These three cell lines (Hela, Chang liver, and Bel-7402) were all transfected with an over-expression vectors of JunD. As shown in Figure 7B, the expression levels of JunD were obviously up-regulated in all three cell lines after transfection. It could also be seen from Figure 7C that the survival rate of cell lines transfected with JunD over-expression vector was significantly increased under T-2 toxin stress compared to wild-type cells. These results showed that JunD played a critical role in the apoptotic process, and its over-expression could effectively prevent the cells from damaging effects by T-2 toxin.

## Discussion

It had been found in many reports that intracellular reduced GSH was down-regulated by T-2 toxin, and at the same time, lipid oxidation occurred and total intracellular ROS was up-regulated [5], [27]. Reduced GSH can effectively remove intracellular free radicals, and the decline in GSH expression level would lead to unbalance of intracellular antioxidant system. The up-regulated ROS and MDA reflected that cells had been coerced by oxidative stress. In the current study, it was clear that all three cell lines (Hela, Bel-7402, and Chang liver) could be induced into the process of apoptosis by T-2 toxin, and the death effect of the toxin against cells was time-dependent and dose-dependent. Oxidative stress might be the main factor which leads to apoptosis under T-2 toxin stress. In the experiment in which antioxidant NAC was used to pretreat cells. The cell line, Hela, was protected efficiently under T-2 toxin stress, but there was no significant effect in Bel-7402 and Chang liver. This may be due to the different sensitivity to NAC for different cell lines. He *et* *al.* (2012) also found that NAC could provide a protective effect towards the apoptosis induced by T-2 toxin [28]. From the reports and results provided above, it could be concluded that oxidative stress plays an important role in apoptosis triggered by T-2 toxin.

Caspase family members are very critical in the process of apoptosis, and they are precisely regulated in the whole process of apoptosis. When they are hydrolyzed by upstream proteases and become activated, they will participate in the process of apoptosis. In the current study, it was suggested that T-2 toxin induced cells apoptosis rather than necrosis by increasing the expression level of Caspase-3 [29]. Caspase-8 plays an important role in death receptor pathway, and Caspase-9 takes part in the mitochondrial pathway [30], [31]. Therefore, The pathway of T-2 toxin induced apoptosis could be illustrated by detecting the protein level of the both proteins. In this study, it was found that the activation of Caspase-9 (about 8 h) was earlier than that of Caspase-8 (about 24 h). So it implied that the apoptosis induced by T-2 toxin was mainly by mitochondrial pathway.

The tumor suppressor p53 plays a very important role in maintaining the integrity of cellular genes and controlling cell growth. So the expression of p53 inside cell is subjected to strict regulation. The apoptosis induced by p53 is through mitochondrial pathway by activating transcription of some pro-apoptotic factors (such as Puma, Bax, p53AIP1, Bcl2, PIGs and so on) [32], [33]. In previous studies of T-2 toxin induced apoptosis, the role of p53 has been controversial. Some studies have reported that p53 does not change in the process [26], whereas some results confirm that p53 is up-regulated in the process [27], [34]. Thus, we found it is important to clarify the role of p53 in T-2 toxin induced apoptosis. It was confirmed in the present study that p53 was up-regulated, which also suggested that the pathway of T-2 induced apoptosis was through the mitochondrial pathway [35].

AP-1 (activator protein-1) transcription factor family plays a key role in the regulation of transcription by Ras which is mainly composed of c-Jun,JunB, and JunD, and it is usually considered that JunD plays an important role in intracellular antioxidant system [36]–[41]. These evidences revealed that there might be an intrinsic link between JunD and oxidative stress, but there is no report about JunD in the study of T-2 toxin induced apoptosis. In our study, we found that JunD was of importance in the apoptotic process induced by T-2 toxin. Initially, we found by Western-blot analysis that the protein levels of JunD were down-regulated in the T-2 toxin induced apoptosis. In further studies on the cell survival rate during T-2 toxin stress, we found that the JunD over-expression cell lines could effectively prevent the effects induced by T-2 toxin. However, there was no obvious difference in the survival rate between wild-type cell lines and JunD interfered cell lines, which might suggest that there are other factors that could complement the function of JunD. Thus, we conclude that JunD could effectively protect cells from T-2 toxin induced apoptosis. It is, however, still unclear how JunD protected the cells from apoptosis under T-2 toxin stress. Further study on the pathway by which JunD prevent apoptosis would illuminate the molecular mechanism of T-2 induced apoptosis.

## References

[1] Bamburg JR, Riggs NV, Strong FM (1968) The structures of toxins from two strains of Fusarium tricinctum. Tetrahedron 24(8): 3329–3336. 5648271 10.1016/s0040-4020(01)92631-6

[2] Yagen B, Bialer M (1993) Metabolism and pharmacokinetics of T-2 toxin and related trichothecenes. Drug Metab Rev 25(3): 281–323. 8404460 10.3109/03602539308993978

[3] Desjardins AE, Hohn TM, McCormick SP (1993) Trichothecene biosynthesis in Fusarium species: chemistry, genetics, and significance. Microbiol Rev 57(3): 595–604. 8246841 10.1128/mr.57.3.595-604.1993 PMC372927

[4] Nelson PE, Dignani MC, Anaissie EJ (1994) Taxonomy, biology, and clinical aspects of Fusarium species. ClinMicrobiology Rev 7(4): 479–504. 10.1128/cmr.7.4.479 PMC358338 7834602

[5] Bouaziz C, Abid-Essefi S, Bouslimi A, EI Golli E, Bacha H (2006) Cytotoxicity and related effects of T-2 toxin on cultured Vero cells. Toxicon 48(3): 343–352. 16884754 10.1016/j.toxicon.2006.06.004

[6] Lutsky I, Mor N (1981) Experimental alimentary toxic aleukia in cats. Lab Anim Sci 31(1): 43–47. 7195959

[7] Magnuson BA (1987) Schiefer HB, Hancock DS, Bhatti AR (1987) Cardiovascular effects of mycotoxin T-2 after topical application in rats. Can J Physiol Pharmacol 65(5): 799–802. 3621042 10.1139/y87-128

[8] Canady RA, Coker RD, Egan SK, Krska R, Olsen M, et al. (2001) T-2 and HT-2 Toxins. WHO Food Additive Series: 47 Safety Evaluation of Certain Mycotoxins in Food. Joint FAO/WHO Expert Committee on Food Additives (JECFA), FAO Food and nutrition paper 74. Available online:http://www.inchem.org/documents/jecfa/jecmono/v47je06.htm.

[9] Bennet JW, Klich M (2003) Mycotoxins. Clin Microbiol Rev 16(3): 497–516. 12857779 10.1128/CMR.16.3.497-516.2003 PMC164220

[10] Thompson WL, Wannemacher RW Jr (1990) In vivo effects of T-2 mycotoxin on synthesis of protein and DNA in rat tissues. Toxicol Appl Pharmacol 105(3): 483–491. 2237920 10.1016/0041-008x(90)90151-j

[11] Chang IM, Mar WC (1988) Effect of T-2 toxin on lipid peroxidation in rats: elevation of conjugated diene formation. Toxicol Letts 40(3): 275–280. 3354011 10.1016/0378-4274(88)90051-3

[12] Islam Z, Nagase M, Yoshizawa T, Yamauchi K, Sakato N (1998) T-2 toxin induces thymic apoptosis in vivo in mice. Toxicol Appl Pharmacol 148(2): 205–214. 9473527 10.1006/taap.1997.8338

[13] Shinozuka J, Li G, Kiatipattanasakul W, Uetsuka K, Nakayama H, et al (1997) T-2 toxin-induced apoptosis in lymphoid organs of mice. Exp Toxicol Pathol 49(5): 387–392. 9455687 10.1016/S0940-2993(97)80124-8

[14] Gavrieli Y, Sherman Y, Ben-Sasson SA (1992) Identification of programmed cell death in situ via specific labeling of nuclear DNA fragmentation. J Cell Biol 119(3): 493–501. 1400587 10.1083/jcb.119.3.493 PMC2289665

[15] Wang X, Liu Q, Ihsan A, Huang L, Dai M, et al (2012) JAK/STAT pathway plays a critical role in the proinflammatory gene expression and apoptosis of RAW264.7 cells induced by trichothecenes as DON and T-2 toxin. Toxicol Sci 127(2): 412–424. 22454431 10.1093/toxsci/kfs106

[16] Stanford GK, Hood RD, Hayes AW (1975) Effect of prenatal administration of T-2 toxin to mice. Res Commun Chem Pathol Pharmacol 10(4): 743–746. 1153850

[17] Williams PP (1989) Effects of T-2 mycotoxin on gastrointestinal tissues: a review of in vivo and in vitro models. Arch Environ Contam Toxicol 18(3): 374–387. 2658861 10.1007/BF01062362 PMC7087545

[18] Mendivil-Perez M, Velez-Pardo C, Jimenez-Del-Rio M (2012) TPEN Induces Apoptosis Independently of Zinc Chelator Activity in a Model of Acute Lymphoblastic Leukemia and Ex Vivo Acute Leukemia Cells through Oxidative Stress and Mitochondria Caspase-3- and AIF-Dependent Pathways. Oxid Med Cell Longev 2012: 313275. 23320127 10.1155/2012/313275 PMC3540963

[19] Martínez-Palacián A, Del Castillo G, Suárez-Causado A, García-Álvaro M, de Morena-Frutos D, et al (2013) Mouse Hepatic Oval Cells Require Met-Dependent PI3K to Impair TGF-β-Induced Oxidative Stress and Apoptosis. PLoS One 8(1): e53108. 23301029 10.1371/journal.pone.0053108 PMC3534654

[20] Gerald D, Berra E, Frapart YM, Chan DA, Giaccia AJ, et al (2004) JunD reduces tumor angiogenesis by protecting cells from oxidative stress. Cell 118(6): 781–794. 15369676 10.1016/j.cell.2004.08.025

[21] Toullec A, Gerald D, Despouy G, Bourachot B, Cardon M, et al (2010) Oxidative stress promotes myofibroblast differentiation and tumour spreading. EMBO Mol Med 2(6): 211–230. 20535745 10.1002/emmm.201000073 PMC3377319

[22] Fang Y, Feng Y, Wu T, Srinivas S, Yang W, et al.. (2013)Aflatoxin B1 Negatively Regulates Wnt/β-Catenin Signaling Pathway through Activating miR-33a. PLoS One 8(8): e73004. Published online 2013 August 27. doi: 10.1371/journal.pone.0073004. 10.1371/journal.pone.0073004 PMC3754916 24015284

[23] Zhuang ZH, Zhao XL, Li H, Wang SY, Peng XX (2011) Gut CaVP is an innate immune protein against bacterial challenge in amphioxus Branchiostoma belcheri. Fish Shellfish Immunol 31(2): 217–223. 21624472 10.1016/j.fsi.2011.05.004

[24] Wu J, Jing L, Yuan H, Peng SQ (2011) T-2 toxin induces apoptosis in ovarian granulosa cells of rats through reactive oxygen species-mediated mitochondrial pathway. Toxicol Lett 202(3): 168–177. 21296132 10.1016/j.toxlet.2011.01.029

[25] Fang H, Wu Y, Guo J, Rong J, Ma L, et al (2012) T-2 toxin induces apoptosis in differentiated murine embryonic stem cells through reactive oxygen species-mediated mitochondrial pathway. Apoptosis 17(8): 895–907. 22614820 10.1007/s10495-012-0724-3

[26] Albarenque SM, Suzuki K, Shinozuka J, Nakayama H, Doi K (2001) Kinetics of apoptosis-related genes mRNA expression in the dorsal skin of hypotrichotic WBN/ILA-ht rats after topical application of T-2 toxin. Exp Toxicol Pathol 52(6): 553–556. 11256758 10.1016/s0940-2993(01)80016-6

[27] Chaudhari M, Jayaraj R, Bhaskar AS, Lakshmana Rao PV (2009) Oxidative stress induction by T-2 toxin causes DNA damage and triggers apoptosis via Caspase pathway in human cervical cancer cells. Toxicology 262(2): 153–161. 19524637 10.1016/j.tox.2009.06.002

[28] He SJ, Hou JF, Dai YY, Zhou ZL, Deng YF (2012) N-acetyl-cysteine protects chicken growth plate chondrocytes from T-2 toxin-induced oxidative stress. Appl Toxicol 32(12): 980–985. 10.1002/jat.1697 21796648

[29] Souza CO, Santoro GF, Figliuolo VR, Nanini HF, de Souza HS, et al (2012) Extracellular ATP induces cell death in human intestinal epithelial cells. Biochim Biophys Acta 1820(12): 1867–1878. 22951220 10.1016/j.bbagen.2012.08.013

[30] Ji Y, Ji C, Yue L, Xu H (2012) Saponins isolated from Asparagus induce apoptosis in human hepatoma cell line HepG2 through amitochondrial-mediated pathway. Curr Oncol 19(Suppl 2): eS 1–9. 10.3747/co.19.1139 PMC3413253 22876162

[31] Tang X, Xing Z, Tang H, Liang L, Zhao M (2011) Human cell-death-inducing DFF45-like effector C induces apoptosis via Caspase-8. Acta Biochim Biophys Sin 43(10): 779–786. 21865223 10.1093/abbs/gmr073

[32] Coureuil M, Ugolin N, Tavernier M, Chevillard S, Barroca V, et al (2010) Puma and Trail/Dr5 pathways control radiation-induced apoptosis in distinct populations of testicular progenitors. PLoS One 5(8): e12134. 20711434 10.1371/journal.pone.0012134 PMC2920820

[33] Szak ST, Mays D, Pietenpol JA (2001) Kinetics of p53 binding to promoter sites in vivo. Mol Cell Biol 21(10): 3375–3386. 11313463 10.1128/MCB.21.10.3375-3386.2001 PMC100259

[34] Chen JH, Cao JL, Chu YL, Wang ZL, Yang ZT, et al (2008) T-2 toxin-induced apoptosis involving Fas, p53, Bcl-xL, Bcl-2, Bax and caspase-3 signaling pathways in human chondrocytes. J Zhejiang Univ Sci B 9(6): 455–463. 18543398 10.1631/jzus.B0820013 PMC2408698

[35] Frank AK, Pietsch EC, Dumont P, Tao J, Murphy ME (2011) Wild-type and mutant p53 proteins interact with mitochondrial caspase-3. Cancer Biol Ther 11(8): 740–745. 21307660 10.4161/cbt.11.8.14906 PMC3100564

[36] Bos JL (1989) ras oncogenes in human cancer: a review. Cancer Res 49(17): 4682–4689. 2547513

[37] Chandel NS, Maltepe E, Goldwasser E, Mathieu CE, Simon MC, et al (1998) Mitochondrial reactive oxygen species trigger hypoxia-induced transcription. Proc. Natl. Acad. Sci. USA 95(20): 11715–11720. 10.1073/pnas.95.20.11715 PMC21706 9751731

[38] Fandrey J, Genius J (2000) Reactive oxygen species as regulators of oxygen dependent gene expression. Adv Exp Med Biol 475: 153–159. 10849657 10.1007/0-306-46825-5_15

[39] Jaakkola P, Mole DR, Tian YM, Wilson MI, Gielbert J, et al (2001) Targeting of HIF- to the von Hippel-Lindau ubiquitylation complex by O_2_-regulated prolyl hydroxylation. Science 292(5516): 468–472. 11292861 10.1126/science.1059796

[40] Lallemand D, Spyrou G, Yaniv M, Pfarr CM (1997) Variations in Jun and Fos protein expression and AP1 activity in cycling, resting, and stimulated fibroblasts. Oncogene 14(7): 819–830. 9047389 10.1038/sj.onc.1200901

[41] Knowles HJ, Raval RR, Harris AL, Ratcliffe PJ (2003) Effect of ascorbate on the activity of hypoxia-inducible factor in cancer cells. Cancer Res 63(8): 1764–1768. 12702559

